# Structural Characterization and Antioxidant Activity of Polysaccharides From Indigo Naturalis *Ophiopogon japonicus*: Effects of Processing and Extraction Methods

**DOI:** 10.1002/fsn3.71522

**Published:** 2026-02-10

**Authors:** Yi Zhao, Yi Zhong, Qi Zheng, Chun‐Yan Yin, Jia‐Li Cai, Di‐Jun Wang, Li Zhu, Ji Cao, Xiao‐Jing Yan, Yuan‐Pei Lian

**Affiliations:** ^1^ Changzhou Key Laboratory of Human Use Experience Research and Transformation of Menghe Medical School Changzhou Hospital of Chinese Medicinal Affiliated to Nanjing University of Chinese Medicine Changzhou P. R. China

**Keywords:** antioxidant activity, functional food, *Indigo naturalis*, *Ophiopogon japonicus*, polysaccharides, structural characterization, traditional processing

## Abstract

*Indigo naturalis‐*processed *Ophiopogon japonicus* (IN‐OJ) is a common processing method in the Menghe medical school to enhance its heat‐clearing and detoxifying effects. However, its impact on the structure and bioactivity of the main active component—polysaccharides—remains unclear. In this study, polysaccharides were extracted from both raw and IN‐processed *O. japonicus* using hot reflux and ultrasound‐assisted methods. Their structural features were characterized by scanning electron microscopy (SEM), transmission electron microscopy (TEM), gel permeation chromatography (GPC), high‐performance liquid chromatography (HPLC), and Fourier‐transform infrared spectroscopy (FT‐IR). Monosaccharide composition, molecular weight, and microscopic morphology were systematically analyzed. Antioxidant activities were evaluated in vitro through DPPH, superoxide anion, and hydroxyl radical scavenging assays, using Vitamin C (Vc) as a positive control. The results indicated that IN processing significantly increased the polysaccharide and uronic acid contents. Molecular weight profiles revealed an additional high‐molecular‐weight fraction in the processed samples (U‐IP: 520,049 kDa; W‐IP: 356,167 kDa). Monosaccharide analysis showed notable increases in arabinose and fucose. Microscopic observations indicated a transition from a loose, honeycomb‐like structure to a denser, aggregated morphology, while FT‐IR spectroscopy confirmed the introduction of additional carboxyl groups. Furthermore, antioxidant assays demonstrated that IN processing significantly enhanced scavenging activities against DPPH and superoxide anion radicals, with W‐IP exhibiting the strongest DPPH radical scavenging capacity (IC_50_ = 3.19 mg/mL). However, hydroxyl radical scavenging activity decreased post‐processing, likely due to steric hindrance within the aggregated structures. The positive control Vc exhibited markedly superior scavenging activity across all assays. These findings suggest that IN processing enhances specific antioxidant capacities of OJ polysaccharides through structural modification, with reflux extraction yielding the most active fractions, thus providing a scientific basis for optimizing this traditional method in functional food applications.

## Graphical abstract

*Indigo naturalis* processing significantly increased polysaccharide and uronic acid content, altered monosaccharide composition, and introduced high‐molecular‐weight fractions. Structural changes enhanced DPPH and superoxide anion scavenging activities, especially in reflux‐extracted processed samples (W‐IP, IC_50_ = 3.19 mg/mL). The study validates the traditional “*Ban‐Zhi*” method and provides a basis for developing functional foods.

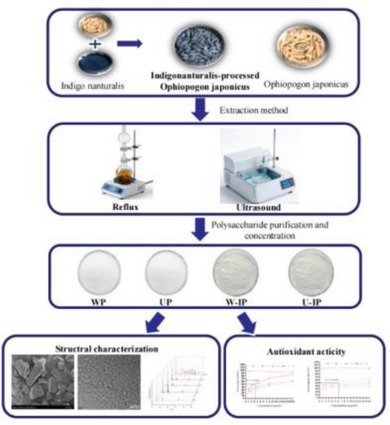

## 1 Introduction

Traditional processing techniques in Chinese medicine play a pivotal role in enhancing the functional activities of natural products. Among these, coating or “*Ban‐Zhi”* methods involve adhering functional excipients to the surface of herbal materials, thereby modulating their physicochemical properties and bioactivities. This approach has attracted increasing attention for its potential to enhance efficacy and reduce adverse effects (Li et al. 2025). *Indigo naturalis‐*processed *Ophiopogon japonicus* (IN‐OJ) represents a classical processing practice in the Menghe medical school, where *O. japonicus* (OJ) is coated with Indigo naturalis (IN) to reinforce its “heat‐clearing and detoxifying” properties and alleviate chest obstruction caused by blood stasis and phlegm dampness. However, the underlying chemical transformations and functional mechanisms involved in this traditional processing remain poorly understood.

*Indigo naturalis* (Qing Dai) is a natural excipient rich in bioactive pigments. Historically documented in the Compendium of Materia Medica for “clearing heat, stopping bleeding, and healing sores,” modern studies have identified key components such as indigo, indirubin, tryptanthrin, and β‐sitosterol (Plitzko et al. 2009; Qi‐Yue et al. 2020), which exhibit antitumor, antiviral, antibacterial, and anti‐inflammatory activities (Liang et al. 2019; Qi‐Yue et al. 2020). *Ophiopogon japonicus* (Mai Dong), a dual‐purpose medicinal and edible plant widely distributed in China, Japan, and Vietnam, is traditionally used for nourishing Yin, generating body fluids, and clearing the heart and lungs. Pharmacological studies have confirmed that OJ exerts anti‐inflammatory, anti‐aging, and antioxidant effects (Chen et al. 2016). Among its bioactive constituents, polysaccharides (OPJ) are abundant and display multiple biological activities, including immunomodulation, antioxidant effects, hypoglycemic effects, cardio protection, and gut microbiota regulation (Fang et al. 2018). A recent comprehensive review has systematically summarized the preparation methods, structural features, and diverse biological activities of *Ophiopogon japonicus* polysaccharides, reinforcing their significance as a key bioactive component (Zhu et al. 2025). Their bioactivities are highly dependent on structural features such as molecular weight, monosaccharide composition, glycosidic linkages, and chain conformation (Yang et al. 2026). For example, polysaccharides rich in arabinose, galactose, and uronic acids often exhibit superior DPPH and superoxide radical scavenging capacities (Wang et al. 2024).

In recent years, the structural transformation of polysaccharides during traditional Chinese medicine processing and its impact on biological activity has gained increasing attention. Shi reported that processing Rahmanian glutinous reduced its polysaccharide molecular weight and increased arabinose content, resulting in enhanced antioxidant and anti‐aging effects (Shi et al. 2025). Xia demonstrated that honey‐processing Glycyrrhiza uralensis elevated the proportion of mannose, rhamnose, and glucuronic acid in its polysaccharides, significantly improving anti‐fatigue activity (Xia et al. 2024). Yao observed that processed Polygona tum polysaccharides exhibited lower molecular weights and increased rhamnose and galactose contents, providing stronger protection in an acute kidney injury mouse model (Yao et al. 2024). Despite these findings, the structural transitions and bioactivity variations of polysaccharides during IN‐OJ processing remain largely unexplored, limiting its broader application in functional foods and nutraceuticals.

Extraction methods are another critical factor determining the structural integrity and functional performance of polysaccharides. Common techniques include hot water extraction, ultrasound‐assisted extraction, microwave‐assisted extraction, and enzyme‐assisted extraction (Lian et al. 2026). Extraction not only affects yield and purity but may also induce chain cleavage or conformational rearrangements that influence antioxidant or immunomodulatory activities (Kakar et al. 2020). Chen found that ultrasound‐extracted polysaccharides from ginger pomace exhibited higher yields and stronger antioxidant activity, possibly due to increased acidic monosaccharide content. (Chen et al. 2019). Ding compared mung bean polysaccharides obtained via different methods and reported that alkali‐extracted polysaccharides demonstrated superior antioxidant activity, whereas hot water extracts were more effective in suppressing LPS‐induced IL‐6 and TNF‐α release in Caco‐2 cells (Ding et al. 2025).

Collectively, these findings suggest that coating processes and extraction strategies synergistically influence the physicochemical and functional properties of polysaccharides in natural products. However, no systematic investigation has yet examined the structural characteristics and antioxidant activities of polysaccharides before and after IN‐OJ processing. Therefore, in this study, we employed both hot reflux and ultrasound‐assisted extraction to compare the structural composition, physicochemical properties, and in vitro antioxidant activities of polysaccharides from raw and IN‐OJ samples. This work aims to elucidate the processing mechanisms and functional implications of traditional combinatorial preparation from a functional food perspective, providing a scientific foundation for the development of modern polysaccharide‐based formulations inspired by traditional processing concepts.

## 2 Materials and Methods

### 2.1 Materials

*Ophiopogon japonicus* and *Indigo naturalis* were purchased from Suzhou Tian ling Traditional Chinese Medicine Decoction Pieces Co. Ltd. (Suzhou, China). Chloroform and sulfuric acid were obtained from Sinopharm Chemical Reagent Co. Ltd. (Shanghai, China). 95% ethanol was purchased from Yongda Reagent Co. Ltd. (Tianjin, China). Anhydrous glucose was supplied by Shanghai Yuan ye Biotechnology Co. Ltd. (Shanghai, China). 2,2‐Diphenyl‐1‐picrylhydrazyl (DPPH), gallic acid and ferrous sulfate were purchased from Macklin Biochemical Co. Ltd. (Shanghai, China). Hydrogen peroxide solution was supplied by Anyan Technology Co. Ltd. (Xiamen, China), and Tris (hydroxymethyl) aminomethane was from Macklin Biochemical Co. Ltd. (Shanghai, China). Standard monosaccharides: mannose (Man), ribose (Rib), rhamnose (Rha), glucuronic acid (GlcA), galacturonic acid (GalA), glucose (Glc), galactose (Gal), xylose (Xyl), arabinose (Ara), and fucose (Fuc) were purchased from the Shanghai Lanji Technology Development Company.

### 2.2 Experimental Methods

#### 2.2.1 Preparation of *Indigo Naturalis*‐Processed *O. japonicus* (IN‐OJ)

100 g fresh *O. japonicus* was moistened with 8 mL of water for 4 min, followed by thorough mixing with 18 g of Indigo naturalis. The mixture was dried at ambient temperature and stored for subsequent experiments.

#### 2.2.2 Extraction of Crude Polysaccharides

Powdered OJ and IN‐OJ (100 g each, passed through a 40‐mesh sieve) were extracted with distilled water at a solid‐to‐liquid ratio of 1:12 (g/mL) using either hot reflux (95°C, 3 h) or ultrasound‐assisted extraction (1500 W, 26°C, 30 min), repeated three times. The combined extracts were filtered and concentrated, followed by precipitation with three volumes of 95% ethanol overnight. The resulting precipitates were collected and assigned as follows: W‐IP: Reflux‐extracted polysaccharides from IN‐OJ; WP: Reflux‐extracted polysaccharides from raw OJ; U‐IP: Ultrasound‐extracted polysaccharides from IN‐OJ; UP: Ultrasound‐extracted polysaccharides from raw OJ.

#### 2.2.3 Purification of Crude Polysaccharides

The crude polysaccharides were dissolved in distilled water and deproteinized using Savage reagent (chloroform: *n*‐butanol = 4:1, v/v) with vigorous shaking for 20 min, followed by centrifugation at 3000 rpm for 10 min. The lower organic phase was removed, and the aqueous phase was retained. This procedure was repeated three times. After organic solvent removal, the aqueous fractions were freeze‐dried to obtain purified polysaccharides.

#### 2.2.4 Structural Characterization of Polysaccharides

##### 2.2.4.1 Polysaccharide Content Analysis

The total polysaccharide content was determined using the phenol‐sulfuric acid method. A glucose standard curve was constructed using concentrations of 4–256 μg/mL (Y = OD_490_ nm vs. X = glucose concentration). Sample solutions (256 μg/mL) were treated according to the same protocol, and total sugar content was calculated from the linear equation.

##### 2.2.4.2 Analysis of Uronic Acid Content

The uronic acid content was determined using the m‐hydroxydiphenyl method. A standard curve was constructed with a concentration range of 0–100 μg/mL (Y = OD_525_ nm vs. X = galacturonic acid concentration). The sample solution (100 μg/mL) was treated using the same method, and the uronic acid content was calculated based on the linear equation.

##### 2.2.4.3 UV–Visible Spectroscopy

Polysaccharides were dissolved in ultrapure water (1 mg/mL), and UV–Vis spectra were recorded over 200–400 nm to detect protein or nucleic acid impurities.

##### 2.2.4.4 Molecular Weight Distribution

Polysaccharides (3–10 mg/mL) were analyzed using a TSK gel GMPWXL aqueous gel column. The mobile phase consisted of 0.1 mol/L NaNO_3_ with 0.05% NaN_3_ at 0.6 mL/min, and molecular weights were determined by calibration with standard dextrans.

##### 2.2.4.5 Scanning Electron Microscopy (SEM) Analysis

A small amount of each polysaccharide sample was evenly adhered onto a carbon conductive adhesive tape by gently touching the tape's white protective paper to the powder. The sample‐loaded tape was then mounted on a specimen stub. The stub was placed approximately 6.9 mm from the evaporation source in a sputter coater. During coating, the stub was rotated to ensure uniform deposition. A thin conductive layer was applied via gold sputtering (3 kV, 60 s). After coating, the samples were observed under the scanning electron microscope.

##### 2.2.4.6 Transmission Electron Microscopy (TEM) Analysis

The polysaccharide sample was first dispersed in deionized water via ultrasonication to obtain a homogeneous suspension. A droplet of the suspension was then carefully deposited onto an ultra‐thin carbon film supported on a copper grid and allowed to air‐dry completely at room temperature. The prepared grid was subsequently examined using a transmission electron microscope.

##### 2.2.4.7 Monosaccharide Composition

Samples were hydrolyzed with 2 mol/L trifluoroacetic acid (TFA) at 120°C for 4 h under nitrogen. After drying under N₂ and reconstitution, hydrolysates were derivatized with 1‐phenyl‐3‐methyl‐5‐pyrazolone (PMP), neutralized, and extracted with chloroform. The supernatant was filtered (0.22 μm) and analyzed by HPLC using an Xtimate C_18_ column (4.6 × 200 mm, 5 μm). The mobile phase was 0.05 mol/L KH₂PO₄ (adjusted to pH 6.7) and acetonitrile (83:17, v/v) at a flow rate of 1 mL/min. Monosaccharide species were identified by comparison with standard mixtures.

##### 2.2.4.8 Fourier Transform Infrared Spectroscopy

Polysaccharides (1.3 mg) were ground with 130 mg KBr, pressed into pellets, and scanned at 4000–400 cm^−1^ to determine characteristic functional groups.

#### 2.2.5 Antioxidant Activity Test

##### 2.2.5.1 DPPH Radical Scavenging Assay

The DPPH radical scavenging assay was performed according to a previously reported method with slight modifications (Lin and Huang 2025). Although the polysaccharides were water‐extracted, the DPPH assay was conducted in an ethanolic system because DPPH is highly stable and soluble in ethanol, and this is a widely established and standardized protocol for evaluating antioxidant capacity. Briefly, polysaccharide solutions (1–16 mg/mL in water) were mixed with an equal volume of 0.1 mmol/L DPPH in ethanol. The mixture was vortexed thoroughly to ensure a homogeneous reaction system and incubated in the dark at room temperature for 30 min. The absorbance was measured at 517 nm. The scavenging rate was calculated as follows:

$$\text{Scavenging rate}\;\left(\%\right)=\left(1-\frac{A_{1}-A_{0}}{A_{2}}\right)\times 100\%$$

##### 2.2.5.2 Superoxide Anion Radical Scavenging Assay

Following the reported method (Zhang et al. 2011; 12(5)), 20 μL of polysaccharide solution (1–16mg/mL) was mixed with 180 μL Tris–HCl buffer (50 mmol/L, pH 8.2) pre‐incubated at 37°C for 20 min, followed by 20 μL 3 mmol/L pyrogallol. Absorbance was measured at 325 nm after immediate mixing, The scavenging rate was calculated according to the general formula provided in section 2.2.5.1.

##### 2.2.5.3 Hydroxyl Radical Scavenging Assay

According to the modified method of Hou (Hou 2017), the reaction system contained 50 μL polysaccharide solution, 50 μL FeSO_4_ (9 mmol/L), 50 μL H₂O₂ (8.8 mmol/L), and 50 μL salicylic acid (9 mmol/L). After incubation at 37°C for 30 min, absorbance was measured at 510 nm. Hydroxyl radical scavenging rate was calculated as:

$$\text{Scavenging rate}\;\left(\%\right)=\left(\frac{A_{0}-A_{1}}{A_{0}}\right)\times 100\%$$

### 2.3 Statistical Analysis

All experiments were performed in triplicate, and results were expressed as mean ± standard deviation (Mean ± SD). Statistical significance was evaluated using one‐way *ANOVA*, and differences were considered significant at *p* < 0.05.

## 3 Results

### 3.1 Physicochemical Characterization of Polysaccharides

#### 3.1.1 Polysaccharide Yield and Content

The yields of the preliminarily purified polysaccharides UP, WP, U‐IP, and W‐IP were 20.15%, 36.50%, 17.20%, and 23.80%, respectively. The glucose standard curve was constructed with the regression equation *Y* = 0.0134X + 0.0363 (*R* ^*2*^ = 0.9996). The polysaccharide contents of each fraction are presented in Table 1. The results indicate that the polysaccharide content of OJ significantly increased after processing with IN, while the extraction method (Ultrasonic vs. Reflux) exerted only a minor influence on polysaccharide content.

**TABLE 1:** The polysaccharide and uronic acid content of UP, WP, U‐IP and W‐IP (%).

Sample	UP	WP	U‐IP	W‐IP
Content				
Total sugars	83.25 ± 0.40	83.92 ± 1.30	88.32 ± 0.50	88.78 ± 1.51
Uronic acid	3.97 ± 0.35	5.15 ± 0.40	5.42 ± 0.31	7.43 ± 1.20

#### 3.1.2 Uronic Acid Content

A regression equation was established using galacturonic acid: Y = 0.0049x + 0.0618 (*R* ^*2*^ = 0.996). The uronic acid content of each polysaccharide component is shown in Table 1. The order of uronic acid content was W‐IP > WP > U‐IP > UP, with specific values of 7.43% ± 1.2%, 5.42% ± 0.31%, 5.15% ± 0.4%, and 3.97% ± 0.35%. The experimental results indicated that the uronic acid content of *Ophiopogon japonicus* (OJ) significantly increased after IN treatment. Among the methods, reflux extraction demonstrated a more significant effect on uronic acid content compared to ultrasonic extraction.

#### 3.1.3 UV Spectroscopy Analysis

**FIGURE 1:**
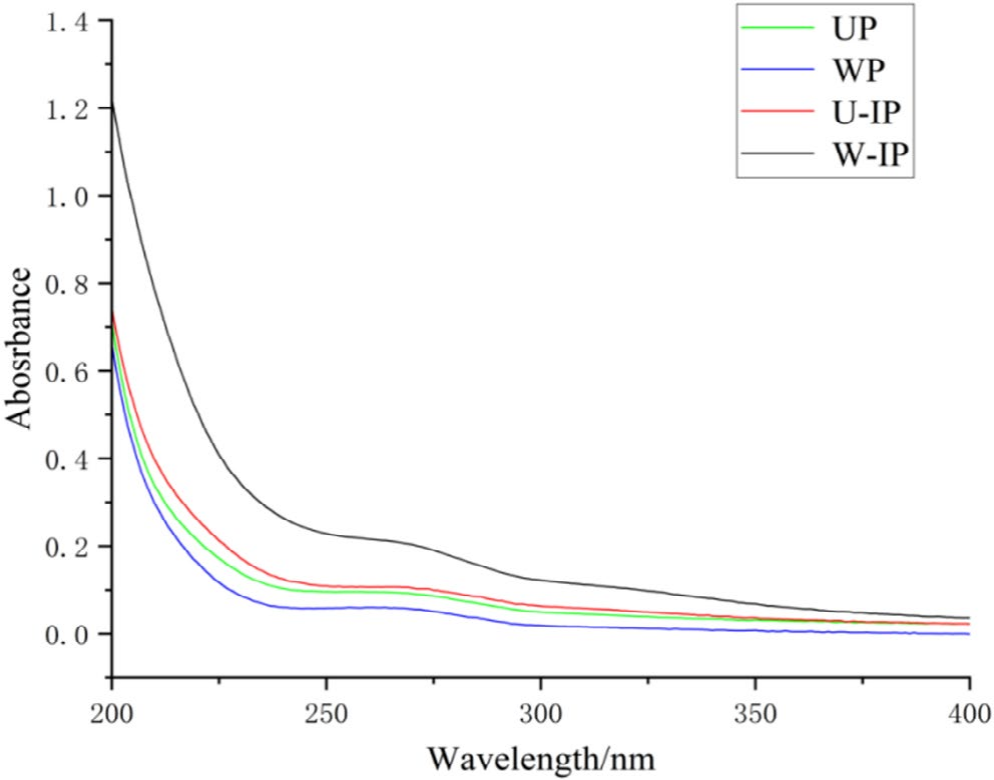
UV spectra of UP, WP, U‐IP, and W‐IP.

As shown in Figure 1, no obvious absorption peaks were observed for the four polysaccharides at 260 or 280 nm, indicating that the preliminarily purified fractions were free of nucleic acid and protein contamination.

#### 3.1.4 Molecular Weight Distribution Analysis

**FIGURE 2:**
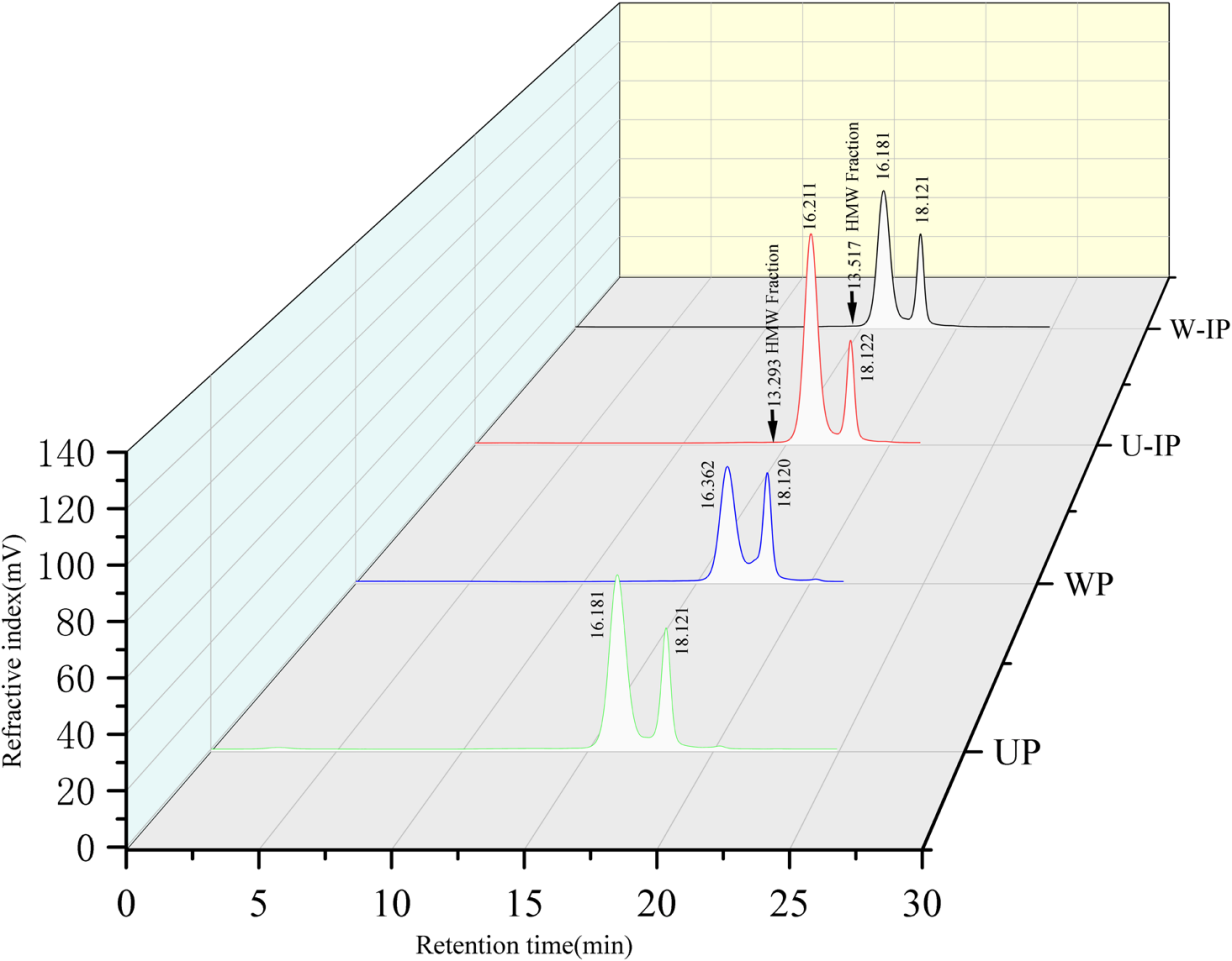
GPC patterns of UP, WP, U‐IP, and W‐IP. Arrows indicate the new high‐molecular‐weight fractions found in the IN‐processed samples (U‐IP and W‐IP).

The molecular weight distributions of the polysaccharides are presented in Figure 2 and Table 2. The GPC profiles of the unprocessed samples (UP and WP) showed two well‐separated and symmetrical peaks. In contrast, the chromatograms of the IN‐processed samples (U‐IP and W‐IP) revealed significant changes. Close examination showed the emergence of shoulder peaks or new populations in the high molecular weight region (as indicated by arrows in Figure 2), which were absent in the unprocessed controls. This visual observation is conclusively supported by the quantitative data in Table 2, which shows the appearance of a discrete, very high molecular weight fraction (Mw > 500,000 Da) in both U‐IP and W‐IP, constituting 0.3%–0.45% of the sample. These results demonstrate that IN processing introduced new polysaccharide species, leading to a more complex and broader molecular weight distribution, likely due to aggregation or the incorporation of IN‐derived macromolecules. Notably, a high‐molecular‐weight fraction was detected, which is likely derived from the intrinsic polysaccharides of IN. For U‐IP, the three molecular weights were 520,049, 3622, and 176 kDa, while for W‐IP they were 356,167, 3689, and 177 kDa.

**TABLE 2:** Molecular weight distribution of UP, WP, U‐IP and W‐IP.

Sample name	Weight average molecular weight (Mw)	Number average molecular weight (Mn)	*Z*‐average molecular weight (Mz)	Molecular weight fraction (%)	PDI (Mw/Mn)
UP	3687	5131	6926	71.23	1.39
	229	265	305	28.77	1.16
WP	2889	4013	6575	62.74	1.39
	243	299	375	37.28	1.23
U‐IP	520,049	1,143,546	2,873,705	0.30	2.20
	3622	5275	11,600	76.84	1.46
	176	252	293	22.85	1.43
W‐IP	356,167	747,077	1,732,128	0.45	2.10
	3689	5584	12,224	70.95	1.51
	177	253	293	28.60	1.43

#### 3.1.5 Scanning Electron Microscopy Analysis

**FIGURE 3:**
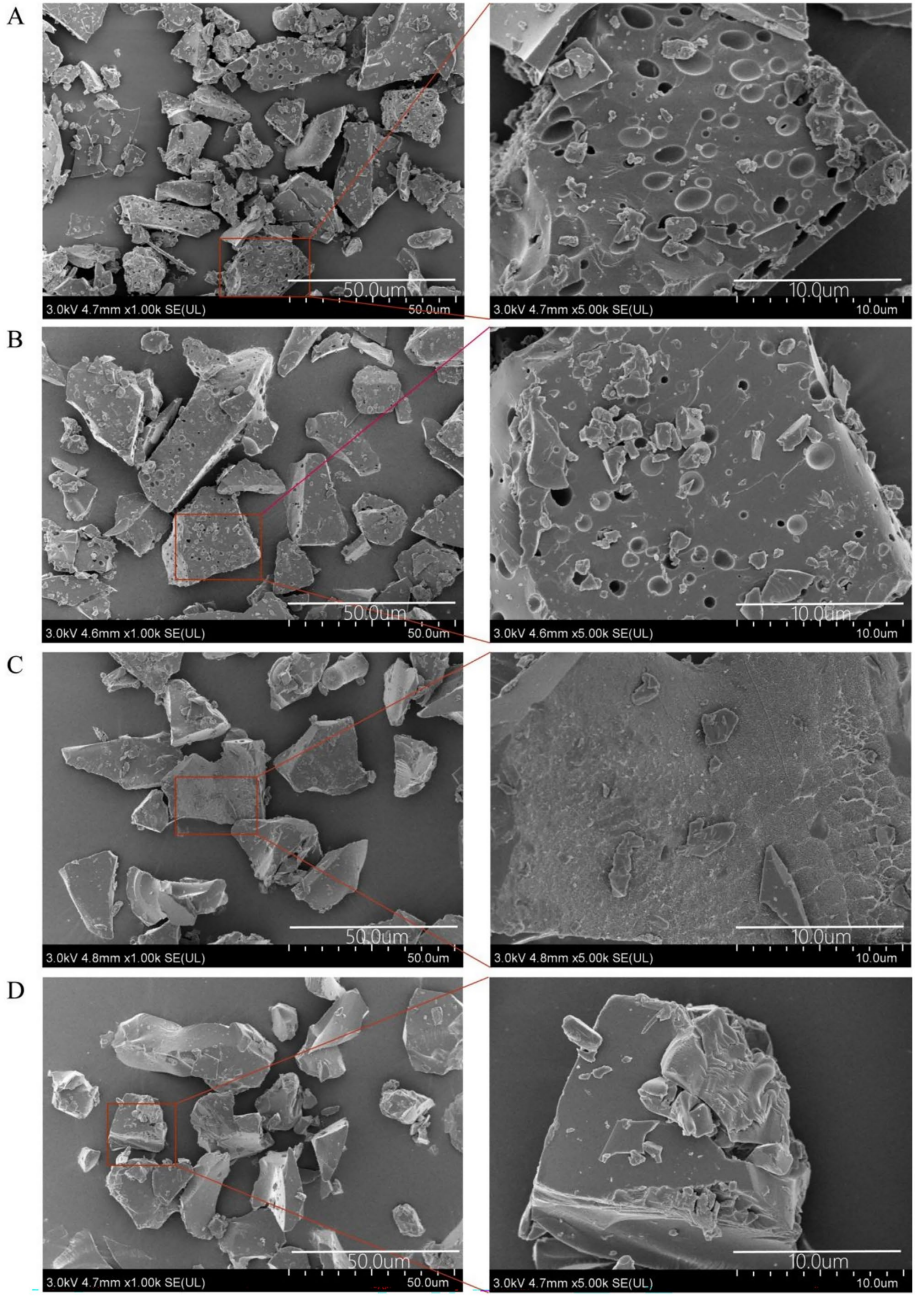
Scanning Electron Microscope (SEM) of UP (A), WP (B), U‐IP (C) and W‐IP (D).

To investigate the effects of different extraction methods on the surface morphology of polysaccharides before and after IN processing, the three‐dimensional structures of the four polysaccharide samples were observed using SEM. As shown in Figure 3, the four samples exhibited marked differences in particle size and surface features. UP and WP (Figure 3A,B) presented fragmented particles with numerous honeycomb‐like cavities on the surface, indicating that their polysaccharide structures were relatively loose and prone to cross‐adhesion. In contrast, U‐IP and W‐IP (Figure 3C,D) displayed more intact, block‐like structures with smoother surfaces and almost no honeycomb‐like pores. This morphology suggests that the polysaccharides in these samples may form more stable aggregates due to tighter interactions with other macromolecular polysaccharides. Additionally, compared with WP, the UP sample exhibited a higher degree of fragmentation and a more pronounced honeycomb‐like morphology, likely resulting from the mechanical shear forces generated during ultrasonic extraction, which disrupt the original aggregated structures of the polysaccharides.

Similarly, among the processed samples, U‐IP displayed a rougher surface than W‐IP, suggesting that even after IN processing, ultrasonic extraction can still cause partial disruption of the polysaccharide microstructure. Overall, these results demonstrate that extraction methods have a pronounced influence on the microstructural morphology of polysaccharides, and that IN processing may partially alleviate structural damage, thereby enhancing the structural stability of the polysaccharides.

#### 3.1.6 Transmission Electron Microscopy Analysis

**FIGURE 4:**
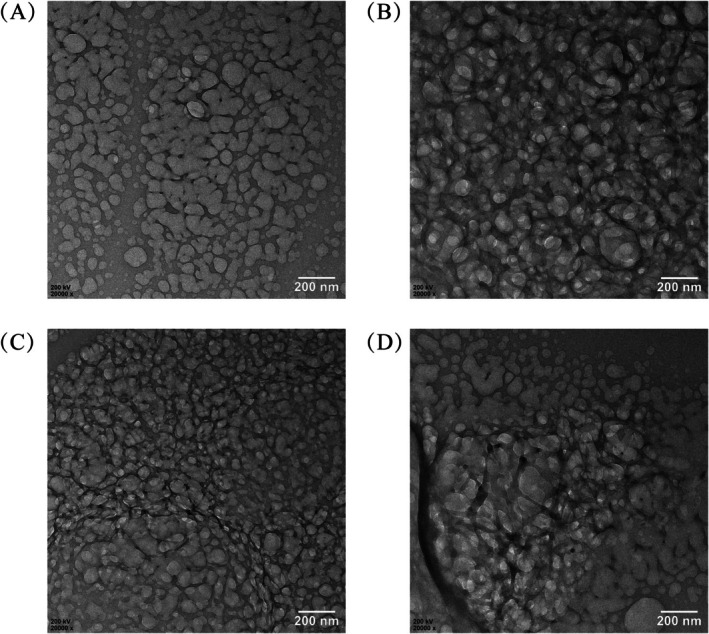
Transmission Electron Microscope (TEM) of UP (A), WP (B), U‐IP (C) and W‐IP (D).

TEM with the results presented in Figure 4 that the polysaccharides obtained before and after IN‐processed OJ exhibited predominantly spherical or ellipsoidal morphologies under both extraction methods. Polysaccharides extracted via ultrasonication appeared more dispersed than those obtained by reflux extraction. Additionally, U‐IP and W‐IP samples displayed multilayered structures with a higher degree of particle aggregation, suggesting that the IN processed step may promote tighter molecular packing among polysaccharide chains. To sum up, the polysaccharides from UP and U‐IP were more dispersed than those from WP and W‐IP, which is likely attributable to the structural alterations induced by ultrasonic extraction on the polysaccharide spatial conformation.

#### 3.1.7 Monosaccharide Composition Analysis

**FIGURE 5:**
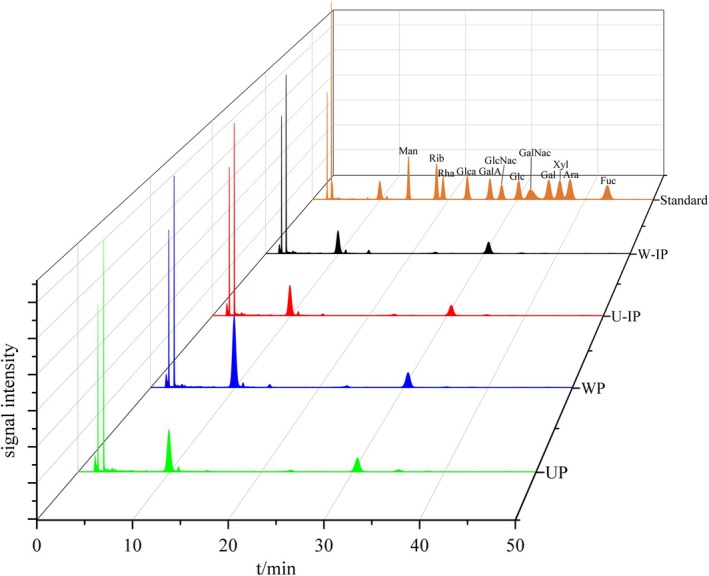
HPLC chromatograms of a monosaccharide mixed standard solution, UP, WP, U‐IP, and W‐IP.

The four polysaccharide fractions were completely hydrolyzed and analyzed for their monosaccharide composition using HPLC, with the results presented in Figure 5 and Table 3. As shown in Figure 5, the chromatographic peaks of 12 monosaccharide standards were well distributed with good resolution, indicating that each monosaccharide could be effectively separated. Comparison with the standard chromatograms revealed that all four polysaccharide samples contained Man, Rib, Rha, GlcA, GalA, Glc, Gal, Xyl, Ara, and Fuc, with glucose being the predominant component, accounting for 73.12%–77.61% of the total. The relative molar percentages of monosaccharides in UP, WP, U‐IP, and W‐IP were as follows: Man: 9.72%, 5.27%, 7.02%, and 2.52%; Rib: 0.36%, 0.48%, 0.37%, and 0.36%; Rha: 0.09%, 0.29%, 0.24%, and 0.71%; GlcA: 8.84%, 10.96%, 8.89%, and 7.18%; GalA: 0.83%, 0.99%, 0.73%, and 0.71%; Gal: 3.97%, 6.32%, 2.94%, and 11.27%; Xyl: 0.20%, 0.36%, 0.25%, and 0.47%; Ara: 1.16%, 1.53%, 1.37%, and 2.73%; Fuc: 0.48%, 0.68%, 0.58%, and 0.85%, respectively.

**TABLE 3:** Monosaccharide composition of UP, WP, U‐IP and W‐IP.

Sample	Amount‐of‐substance percent (%)									
	Man	Rib	Rha	GlcA	GalA	Glc	Gal	Xyl	Ara	Fuc
UP	9.72	0.36	0.09	8.84	0.83	74.36	3.97	0.20	1.16	0.48
WP	5.27	0.48	0.29	10.96	0.99	73.12	6.32	0.36	1.53	0.68
U‐IP	7.03	0.37	0.24	8.89	0.73	77.61	2.94	0.25	1.37	0.58
W‐IP	2.52	0.36	0.72	7.18	0.71	74.18	11.27	0.47	2.73	0.85

#### 3.1.8 Fourier‐Transform Infrared Spectroscopy Analysis

**FIGURE 6:**
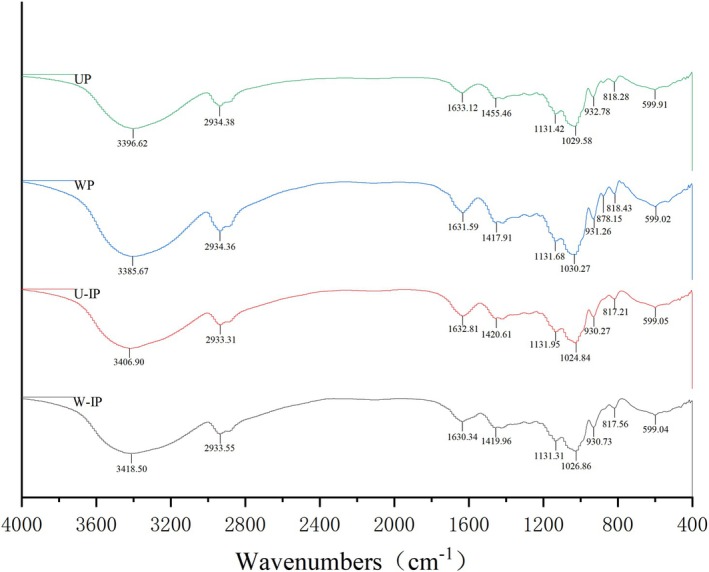
FT‐IR spectra of UP, WP, U‐IP, and W‐IP.

FT‐IR spectroscopy was performed for the four polysaccharide samples in the wavenumber range of 4000–400 cm^−1^, and the spectra are presented in Figure 6. Typically, the IR absorption profiles of the four polysaccharides showed similar peak shapes and intensities, suggesting minimal differences in their main backbone structures. A prominent absorption peak observed around 2934 cm^−1^ corresponded to the C‐H stretching vibrations (Zhang et al. 2024). The absorption peak at approximately 1420 cm^−1^ typically represents the symmetric stretching of carboxylate groups (Su and Li 2020). Close examination of the FT‐IR spectra revealed a consistent enhancement in the absorption band around 1420 cm^−1^ for U‐IP and W‐IP compared to their unprocessed counterparts (UP and WP), as indicated by the arrow in Figure 6. This spectral change, while subtle, aligns with the increase in uronic acid content (Table 2) and supports the hypothesis that IN processing may introduce additional carboxyl groups, indicating that IN processing may have introduced additional carboxyl groups. Combined with the monosaccharide composition results, the elevated levels of Glc and Man in the IN—series polysaccharides suggest that partial oxidation of neutral sugars may have occurred during processing, leading to the formation of new carboxyl groups. This newly introduced carboxyl groups, primarily in ionic form, accounted for the enhanced absorption at 1420 cm^−1^. Notable C—O stretching vibration peaks were also detected around 1131 and 1030 cm^−1^ (Nikonenko et al. 2000), characteristic of ether linkages in the polysaccharide structure. The peak near 1631 cm^−1^ corresponded to C=O stretching, indicating the presence of carbonyl‐containing functional groups such as ketones or aldehydes (Li et al. 2021). Additionally, the absorption peak around 931 cm^−1^ was attributed to the symmetric stretching of fructofuranose rings, and the peak at 818 cm^−1^ to the C‐H bending vibration of fructofuranose methylene groups. A weak band at approximately 875 cm^−1^ likely corresponded to the presence of pyranose glucans and β‐type glycosidic linkages.

As a result, the FT‐IR results were consistent with the monosaccharide composition analysis. In particular, the introduction of additional carboxyl groups further confirmed the structural modification of OJ polysaccharides induced by IN processing. The increase in carboxyl content may enhance the polarity and the number of reactive sites of the polysaccharides, thereby improving their free radical scavenging capacity and antioxidant activity (Liu, Yang, et al. 2025). Analysis of the compositional changes indicated that IN processing of OJ polysaccharides led to a marked increase in the relative contents of Glc, Rha, Xyl, Ara, and Fuc. Notably, W‐IP exhibited a substantial increase in Gal, which became the second most abundant monosaccharide after glucose. Previous studies have reported that elevated levels of Ara and other neutral sugars may be closely associated with enhanced antioxidant activity of polysaccharides (Zhu et al. 2022), suggesting that IN processing may play a potential role in improving the bioactivity of OJ polysaccharides.

### 3.2 Antioxidant Activity Analysis

#### 3.2.1 DPPH Radical Scavenging Assay

**FIGURE 7:**
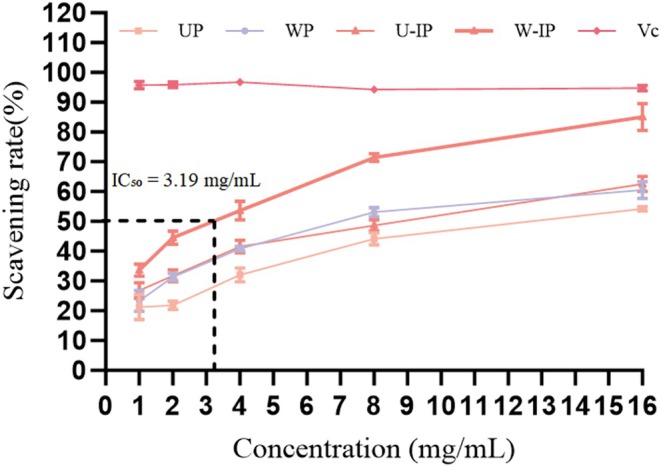
DPPH free radical scavenging rates of UP, WP, U‐IP, W‐IP, and the positive control Vitamin C (Vc).

As shown in Figure 7, the DPPH scavenging rates of the four polysaccharide samples increased with concentration in a dose‐dependent manner. The scavenging efficacy ranked as follows: W‐IP > U‐IP > WP > UP, which was consistent with the trend observed for their IC_50_ values (Table 4). W‐IP exhibited the lowest IC_50_ value (3.19 mg/mL), indicating the strongest DPPH radical scavenging activity, whereas UP showed the highest IC_50_ (12.62 mg/mL), corresponding to the weakest activity. The IC_50_ values of WP and U‐IP were 6.98 and 8.78 mg/mL, respectively. Notably, W‐IP reached a scavenging rate of 85.87% ± 0.63% at 8 mg/mL, while the other samples required a higher concentration (16 mg/mL) to achieve their maximum scavenging rates (W‐IP: 85.04% ± 4.70%; WP: 62.51% ± 2.85%; UP: 54.28% ± 0.84%). All values remained lower than that of the positive control (Vc, IC_50_ < 1 mg/mL). These results clearly demonstrate that IN processing significantly enhances the DPPH radical scavenging capacity of OJ polysaccharides, with reflux extraction combined with IN processing (W‐IP) producing the most potent antioxidant polysaccharides.

**TABLE 4:** IC_50_ values of OJ polysaccharides and Vc in DPPH radical scavenging assay (mg/mL).

Sample	UP	WP	U‐IP	W‐IP	Vc
IC_50_	12.65	6.98	8.78	3.19	< 1.00

#### 3.2.2 Superoxide Anion Radical Scavenging Assay

**FIGURE 8:**
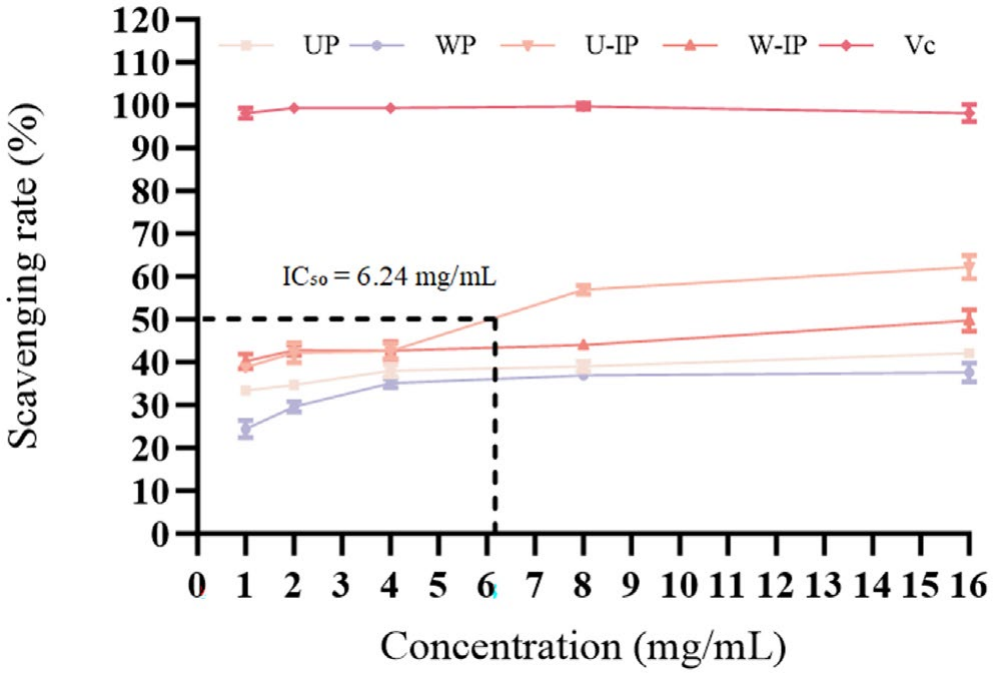
Superoxide anion radical scavenging rates of UP, WP, U‐IP, W‐IP, and the positive control Vitamin C (Vc).

As illustrated in Figure 8, all four polysaccharides exhibited measurable superoxide scavenging activity, which increased in a dose‐dependent manner. The scavenging efficacy ranked as U‐IP > W‐IP > UP > WP, indicating that IN processing markedly enhanced the superoxide radical scavenging performance of OJ polysaccharides. Among these, the ultrasound‐extracted and processed sample (U‐IP) showed the strongest activity. In stark contrast, the positive control Vc exhibited nearly complete scavenging (96%) across all tested concentrations (1–16 mg/mL), demonstrating the exceptionally high sensitivity of this assay to classic antioxidants. At 16 mg/mL, all polysaccharide samples reached their peak scavenging rates, with U‐IP and W‐IP achieving 62.29% ± 2.73% and 49.74% ± 2.87%, respectively. These findings further confirm that IN processing enhances the antioxidant potential of polysaccharides.

#### 3.2.3 Hydroxyl Radical Scavenging Assay

**FIGURE 9:**
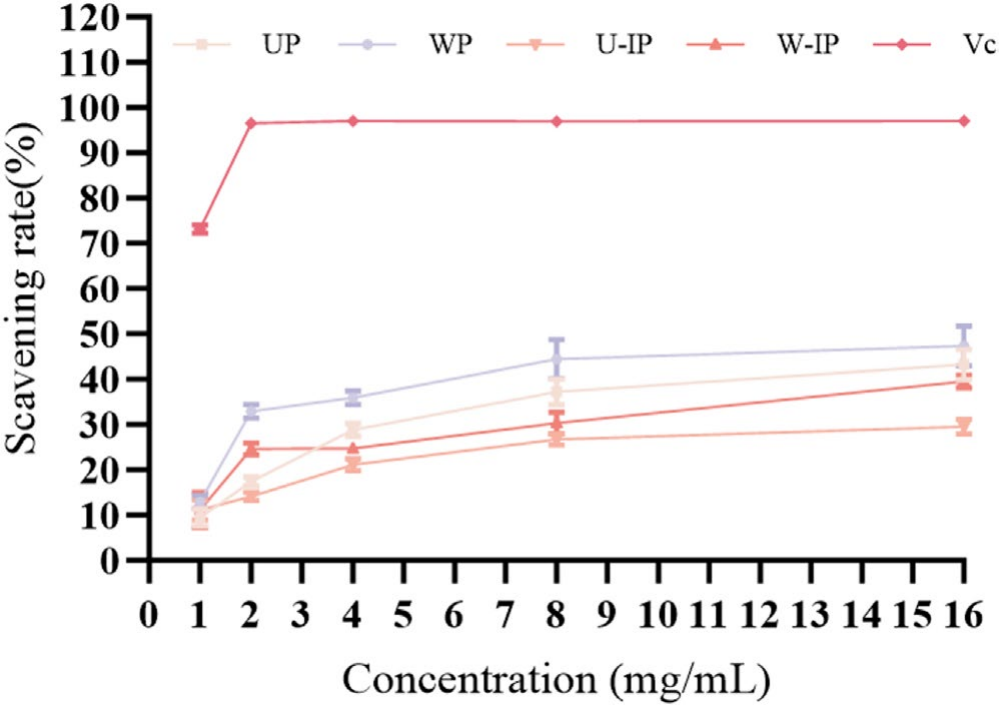
Hydroxyl radical scavenging rates of UP, WP, U‐IP, W‐IP, and the positive control Vitamin C (Vc).

The hydroxyl radical (·OH) is among the most reactive free radicals, capable of rapidly attacking proteins, lipids, and DNA, causing severe cellular and tissue damage. Effective hydroxyl radical scavenging is thus crucial for maintaining oxidative balance in biological and food systems. As shown in Figure 9, the hydroxyl radical scavenging capacities of the four polysaccharides peaked at a concentration of 16 mg/mL, following the order: WP > UP > W‐IP > U‐IP, with corresponding maximum scavenging rates of 47.34% ± 4.62%, 43.28% ± 3.76%, 39.71% ± 1.68%, and 29.53% ± 1.66%. However, the positive control Vc displayed dramatically higher scavenging activity, reaching over 72% at 1 mg/mL and nearly 97% at 2 mg/mL and higher concentrations. This substantial difference underscores the potent efficacy of Vc and positions the polysaccharides as moderate hydroxyl radical scavengers under the tested conditions. Furthermore, the hydroxyl radical scavenging activity decreased after IN processing, despite the overall increase in uronic acid content and the introduction of additional carboxyl groups as evidenced by FT‐IR. This apparent discrepancy may be attributed to the dense aggregation and structural reorganization induced by IN processing, as observed in SEM and TEM analyses. The compact morphology could impose steric hindrance, limiting the diffusion and accessibility of highly reactive ·OH radicals (half‐life < 1 ns) to internal antioxidant sites, thereby reducing scavenging efficiency even in the presence of higher carboxyl group content.

## 4 Discussion

Polysaccharides have garnered significant research interest due to their diverse biological activities, which are intrinsically linked to their complex structural features. The structure–activity relationship is a central theme in polysaccharide research, where parameters such as molecular weight, monosaccharide composition, glycosidic linkage patterns, chain conformation, and higher‐order aggregation states collectively determine their functional performance (Zeng et al. 2019). Both processing methods (traditional herbal processing) and extraction techniques are well known to induce profound structural alterations in polysaccharides, thereby modulating their bioactivity (Kakar et al. 2020; Tian et al. 2024). This study systematically investigated the impact of a specific traditional processing method and two different extraction methods on the structural properties and in vitro antioxidant activities of polysaccharides from *Ophiopogon japonicus* .

The molecular weight profile of polysaccharides is a critical structural determinant influencing their solubility, diffusivity, and interaction with biological targets or free radicals. In this study, IN‐processing dramatically altered the Mw distribution. While raw OJ polysaccharides (UP, WP) showed bimodal distributions, IN‐processed samples (U‐IP, W‐IP) exhibited a tri‐modal profile, featuring a newly appeared, very high molecular weight (HMW) fraction (U‐IP: 520049 kDa; W‐IP: 356167 kDa). This HMW component, likely originating from intrinsic polysaccharides within Indigo naturalis itself or resulting from processing‐induced aggregation, signifies a fundamental “structural reconstruction.” The impact of Mw on antioxidant activity is complex and context‐dependent. For instance, literature often suggests that lower Mw polysaccharides may possess superior antioxidant capacity, attributed to better solubility and increased exposure of reactive groups (Jiang et al. 2011). However, contrasting reports exist, where certain HMW polysaccharides exhibit strong activity attributed to stable conformations that can effectively encapsulate radicals or present a high density of functional groups. Our results present an intriguing scenario: despite the introduction of a HMW fraction, the IN‐processed polysaccharides (particularly W‐IP) demonstrated significantly enhanced scavenging activities against DPPH and superoxide anion (O_2_^−^·) radicals. This suggests that in our system, the potential negative effect of increased Mw on mobility was outweighed by other positive structural modifications induced by processing. This aligns with findings by (Yao et al. 2024), where processed Polygonatum polysaccharides with altered Mw showed enhanced protective effects in vivo, and (Shi et al. 2025), who reported that processing‐induced Mw reduction and compositional changes in Rehmannia glutinosa polysaccharides led to improved anti‐aging activity. Our study adds a new dimension by showing that the introduction of a HMW component, when combined with other favorable changes, can also yield a net positive effect on specific antioxidant pathways.

Beyond Mw, the monosaccharide composition is equally pivotal for bioactivity. Our HPLC analysis revealed that IN‐processing significantly shifted the monosaccharide profile. Notable increases in the relative content of arabinose and fucose were observed across processed samples. Most strikingly, W‐IP showed a substantial increase in galactose, making it the second most abundant sugar. These compositional shifts are highly significant. Numerous studies correlate elevated levels of neutral sugars like arabinose and galactose with enhanced antioxidant capacity in polysaccharides (Liu et al. 2022; Wang et al. 2024). For instance, Huang et al. linked higher Ara content in longan pulp polysaccharides to improved bioactivity (Huang et al. 2019). Therefore, the increase in these specific sugars could influence chain branching, flexibility, and the spatial arrangement of hydroxyl groups, potentially improving hydrogen‐donating capacity and metal chelation—key mechanisms in free radical scavenging (Chou et al. 2019). The FT‐IR results and uronic acid analysis further supported this structural enhancement, confirming the introduction of additional carboxylate groups and an increased uronic acid content in processed samples. Carboxyl and uronic acid groups are potent electron donors and metal chelators, significantly contributing to antioxidant potential (Fernandes and Coimbra 2023; Xue et al. 2025). Furthermore, the increased levels of arabinose, galactose, and fucose, along with the elevated content of carboxyl/uronic groups, had come together to potentially form a polysaccharide structure rich in antioxidant‐active groups, which had effectively accounted for the enhanced DPPH and O_2_^−^ radical‐scavenging capacities observed in our study (Roberto et al. 2024).

The role of extraction methods as a secondary modulator of structure AND function cannot be overlooked. Consistent with findings by (Chen et al. 2019) on ginger pomace and (Ding et al. 2025) on mung bean polysaccharides, our study confirmed that extraction techniques significantly impact polysaccharide characteristics. Hot reflux extraction generally yielded higher polysaccharide and uronic acid content and resulted in a denser, more aggregated morphology (W‐IP) as seen in SEM/TEM. In contrast, ultrasound extraction, known for its mechanical shear effects, produced polysaccharides with a more fragmented, porous morphology (UP, U‐IP) and slightly different Mw profiles, likely due to partial chain cleavage. This structural difference translated into functional divergence: while U‐IP excelled in superoxide anion scavenging, W‐IP was superior in DPPH radical scavenging, achieving the lowest IC_50_ (3.19 mg/mL). This suggests that the more intact, aggregated structure of W‐IP, possibly preserving a more favorable spatial arrangement of functional groups from both OJ and IN, is optimal for electron‐transfer‐based assays like DPPH. However, this very aggregation also provides a plausible explanation for the observed decrease in hydroxyl radical scavenging activity post‐processing. The ·OH radical is extremely reactive and short‐lived, making its scavenging highly dependent on the rapid diffusion and accessibility of antioxidant sites. We hypothesize that the denser, more compact morphology of IN‐processed polysaccharides (U‐IP, W‐IP) creates steric hindrance, limiting the access of·OH radicals to internal reactive groups, despite their higher total content (Kinjo and Takada 2002). This morphology‐dependent accessibility outweighs the benefit of increased functional groups for this specific, diffusion‐limited reaction, highlighting that the structure–activity relationship is mechanism‐specific.

In conclusion, by integrating the influences of traditional IN‐processing and modern extraction techniques, this study elucidates a multifaceted structure–activity relationship. The optimal outcome for producing OJ polysaccharides with potent, broad‐spectrum antioxidant activity was achieved through the synergistic combination of IN‐processing followed by hot reflux extraction, which yielded W‐IP. This protocol successfully integrated the beneficial structural modifications from processing, such as the introduction of new functional components, a favorable shift in monosaccharide composition notably characterized by increased galactose, arabinose, and fucose, and elevated carboxyl/uronic acid content, with an extraction method that preserved a functionally advantageous morphology. Our findings experimentally validate and provide a mechanistic basis for the traditional “Ban‐Zhi” method, highlighting its potential to create structurally and functionally enhanced polysaccharides. These IN‐processed OJ polysaccharides, especially W‐IP, hold promising potential as natural antioxidant ingredients for functional foods and nutraceuticals aimed at mitigating oxidative stress. Future work should focus on the isolation and detailed structural elucidation of the specific active fractions within W1‐IP and the evaluation of its antioxidant efficacy in cellular and in vivo models of oxidative stress.

## 5 Conclusions

In summary, our study demonstrates that the IN‐blending process induces a potential “structural reconstruction” of OJ polysaccharides, while the extraction method further modulates the efficiency of structural release. These findings indicate that IN‐processed OJ polysaccharides, especially the reflux‐extracted W‐IP, demonstrate potential as natural antioxidants in functional foods or supplements to mitigate oxidative stress‐related chronic diseases. The traditional processing method thus demonstrates tangible potential for modern health applications. The combined influence of traditional processing and extraction techniques exerts a significant synergistic effect on the formation of polysaccharide bioactivity, effectively enhancing the yield and improving the antioxidant capacity of OJ polysaccharides. This study identifies reflux extraction combined with IN processing W‐IP as the optimal method for producing antioxidant polysaccharides from IN‐OJ. Our findings experimentally validate the modern application value of the traditional *Ban‐Zhi* method and provide a scientific basis for optimizing extraction procedures in functional TCM‐derived foods. Future studies could further employ advanced structural characterization techniques and cellular oxidative stress models to elucidate the underlying mechanisms, thereby supporting the development of functional foods and modern TCM compound formulations.

## Author Contributions

Y.‐P.L., X.‐J.Y.: investigation (lead), project administration (supporting), supervision (supporting): conceptualization (lead), investigation (lead), project administration (supporting), supervision (supporting). Y.Z., Y.Z.: data curation (supporting), formal analysis (supporting), investigation (supporting), methodology (supporting), supervision (lead), visualization (supporting), writing – review and editing (supporting). Q.Z., C.‐Y.Y., J.‐L.C., D.‐J.W., L.Z., J.C.: resources (supporting). methodology (supporting) and editing (supporting).

## Funding

This work was supported by National Natural Science Foundation of China, 82074016. Jiangsu Provincial Traditional Chinese Medicine Science and Technology Development Plan ‐ Young Talent Project, No. QN202220. Jiangsu Provincial Double Initiative Project, No. JSSCBS20230447. Changzhou Municipal Health Commission, NO. ZD202345. 2024 Postgraduate Research & Practice Innovation Program of Jiangsu Province, SJCX24_0916.

## Ethics Statement

The authors have nothing to report.

## Conflicts of Interest

The authors declare no conflicts of interest.

## Data Availability

The data that support the findings of this study are available from the corresponding author upon reasonable request.
